# Evaluating Conferences as a Tool to Improve Medical Artificial Intelligence Comprehension among Healthcare Professionals: A before and after Study

**DOI:** 10.31662/jmaj.2025-0175

**Published:** 2025-09-26

**Authors:** Nafisa Islam, Abdel Rahman Osman, Laveinia Godfrey

**Affiliations:** 1 Barking, Havering and Redbridge University Hospitals NHS Trust Queen’s Hospital, Romford, United Kingdom; 2 Sherwood Forest Hospitals NHS Foundation Trust King’s Mill Hospital, Sutton in Ashfield, Nottinghamshire, United Kingdom

**Keywords:** medical AI, AI comprehension, AI curriculum, medical education, conference, AI

## Abstract

**Introduction:**

The integration of artificial intelligence (AI) in the United Kingdom’s National Health Service may enhance patient care and alleviate systemic pressures. However, adoption of medical AI is challenged by limited educational access, low confidence among staff, and concerns regarding transparency and ethics. We evaluated the National Health Service National AI Conference on its impact on the understanding and attitudes of health care professionals regarding AI.

**Methods:**

A before-and-after study design was employed using anonymized surveys distributed to conference attendees. The survey assessed participants’ roles, prior experience with medical AI, and perceptions of AI’s risks, benefits, and applications. Using a 5-point Likert scale, responses were analyzed via Wilcoxon’s signed-rank test, after trialing McNemar’s test, with statistical significance defined as p < 0.05.

**Results:**

The survey was completed by 43 attendees. Most were clinical professionals (53.49%), with 65.12% having never attended a similar conference. Most participants (pre-conference: 69.77% vs. post-conference: 85.05%, p = 0.0868) understood the benefits and uses of AI, agreed that AI has the potential to improve patient care (93.02% vs. 95.35%, p = 1.00), were interested in pursuing a career in medical AI (62.79% vs. 67.44%, p = 0.824), and were concerned about the use of AI in health care (65.12% vs. 53.49%, p = 0.419). We observed an increase in understanding of AI after the conference among participants (p = 0.0367). Participant confidence and empowerment increased from 53.49% to 69.77% (p = 0.00319) and from 51.16% to 67.44% (p = 0.00596), respectively; these increases, alongside the increase in understanding of AI, reached statistical significance when analyzed using Wilcoxon’s test, but not when dichotomized and analyzed with McNemar’s test.

**Conclusions:**

Our conference may increase AI understanding, confidence, and empowerment among health care professionals, encouraging further research into targeted medical AI education. A national AI curriculum, transparent governance, and robust information technology infrastructure are recommended to support the adoption of AI internationally.

## Introduction

Internationally, lapses in safety affect one in 10 patients and are responsible for at least three million deaths annually ^(1)^. In the United Kingdom (UK), where health care is delivered free at the point of care through a taxpayer-funded service called the National Health Service (NHS) ^(2)^, human error serves as a significant driver for avoidable mortality and ballooning health care costs. Indeed, clinical negligence complaints have almost doubled over a decade and consist of 1.7% of the total NHS budget ^(3)^. Moreover, while recognized for the high-quality, patient-centered care delivered by its staff, the NHS has experienced declining productivity with a 15-fold increase in those waiting over a year for a procedure in 2024 compared to 2010 ^(3)^.

Artificial intelligence (AI), which can be practically understood as the capacity for machines to approximate, reach, or exceed abilities typically associated with human intelligence ^(4)^, offers a potential solution to this global issue by augmenting human abilities. The World Health Organization (WHO) has highlighted the potential for AI to mitigate health challenges ^(5)^. For example, the use of medical AI to report radiological imaging ^(6)^, complete clinical documentation, and prevent infectious disease ^(7)^ may improve efficiency, accuracy, affordability, and ultimately, patient care ^(6)^.

As health care increasingly embraces AI, it is crucial to focus on educating the workforce most impacted by these changes, including doctors, nurses, pharmacists, and administrative staff ^(8)^. A systematic review by Ahmed et al. ^(9)^ emphasized that the absence of AI education among a health care workforce of 60 was a barrier to its adoption. Furthermore, a systematic review by Gazquez-Garcia et al. ^(10)^ highlighted five vital competencies for health care professionals to effectively implement medical AI. These included fundamentals, ethics, data analysis, communication, and evaluation ^(10)^. According to a report from the Organization for Economic Co-operation and Development, while 72% of doctors recognize AI’s transformative potential, most remain hesitant to integrate it due to concerns around understanding its risks and capabilities ^(11)^. A comprehensive understanding of AI fundamentals may reduce fear among professionals and limit hesitation ^(12)^. Training health care workers on using AI is further highlighted by the WHO to enhance cybersecurity, mitigate bias, and increase patient use ^(5)^.

Current applications of medical AI education are often constrained by limited access to experts in one location, making it difficult for those interested to gain direct exposure to cutting-edge technology. Additionally, there is a lack of awareness and funding, as AI technologies tend to be expensive, and hands-on experiences often require large-scale events with significant financial support. These barriers can hinder the widespread adoption of AI in medical training and education. Conferences may develop AI literacy among NHS professionals, providing the skills and knowledge to evaluate its benefits with the necessary caution required for safe and ethical implementation. The Global Innovation and New Technology (GIANT) Health conference is one of many health care conferences around the UK and the world that facilitates education and networking among professionals interested in the fields of medicine, technology, business, and investment ^(13)^.

Therefore, we analyzed the impact of the GIANT Health conference through a before-and-after study to examine how AI conferences affect the understanding of AI among NHS professionals. Our aims included developing AI comprehension and evaluating sustainable components for an AI curriculum in the NHS.

## Materials and Methods

**Figure 1.**
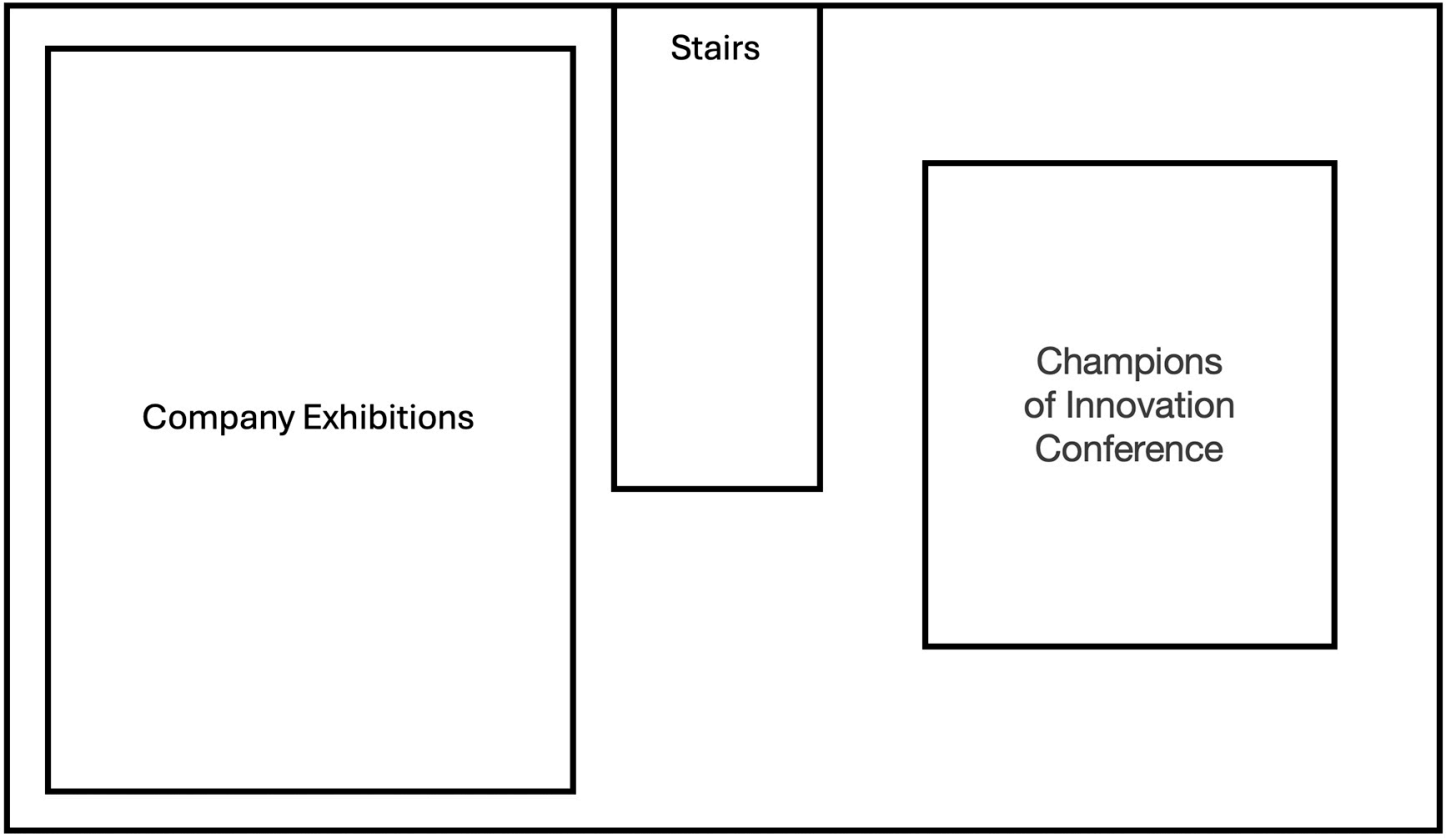
Set-up for the ground floor of the GIANT Health event. Health technology companies exhibited their innovations on one side of the ground floor. The “Champions for Innovation” Conference was delivered on Stage A on the other side. GIANT: Global Innovation and New Technology.

**Figure 2.**
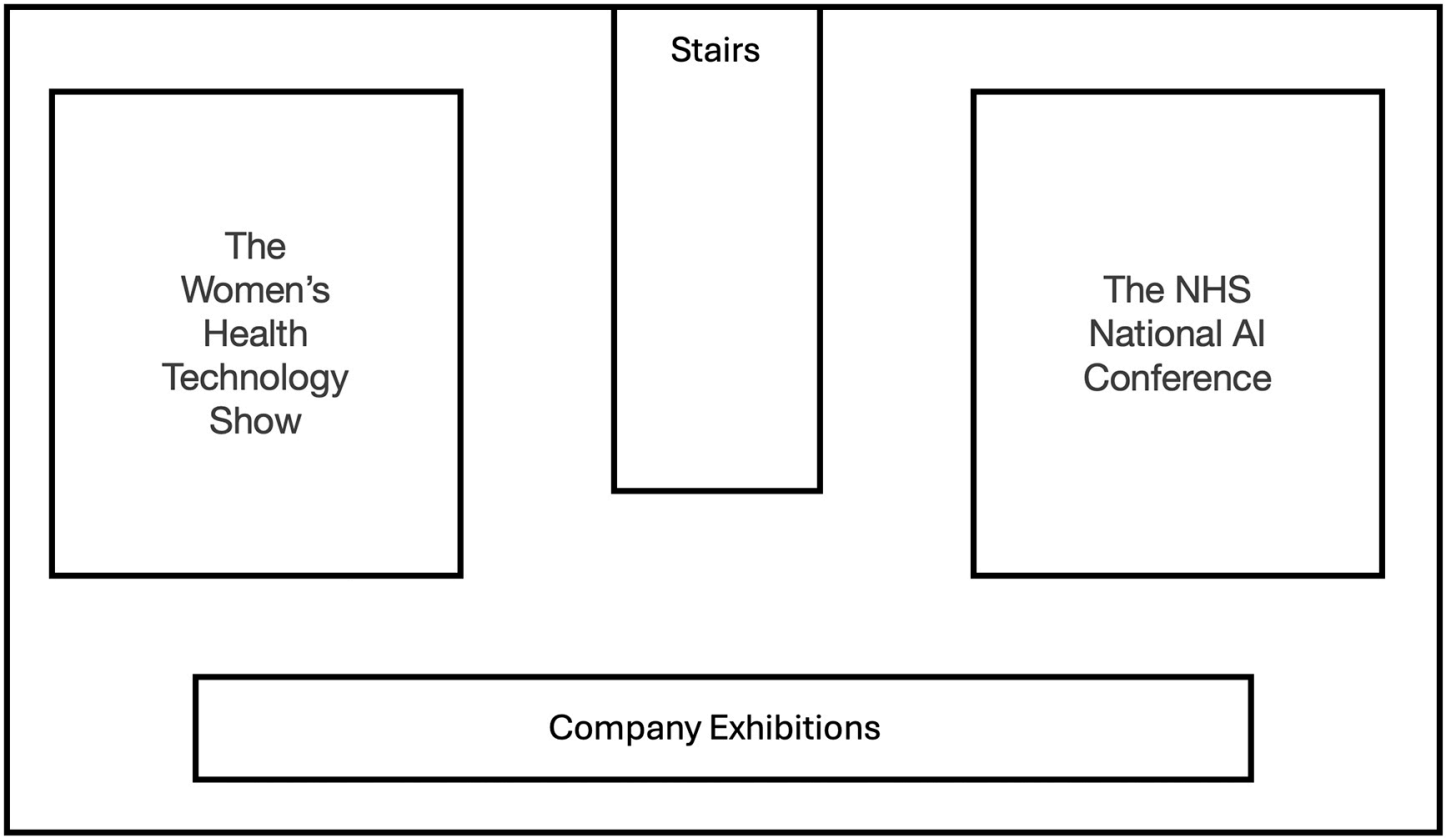
Set-up for the first floor of the GIANT Health event. Health technology companies exhibited their innovations toward the back of the first floor. The “Women’s Health Technology” Conference was delivered on Stage B on one side. The “NHS National AI Conference” was delivered on Stage C. AI: artificial intelligence; GIANT: Global Innovation and New Technology; NHS: National Health Service.

The GIANT Health event has been labeled as “Europe’s largest, most valuable annual festival of health-tech innovation” ^(14)^, typically occurring over two days, with three conferences simultaneously held on each day. On December 10, 2024, the NHS National AI Conference was held alongside two other conferences. Attendees were encouraged to observe the scheduled speakers for all three conferences on the day. Each conference was delivered at a different stage in the same location, so attendees could easily travel between conferences. Figures 1 and 2 demonstrate the layout of the event. Alongside the three conferences, health technology companies exhibited their services and innovations at several stalls. This provided an opportunity for attendees to engage in hands-on activities with AI tools. The GIANT Health event was attended by a variety of people, including health care professionals, start-up businesses, venture capitalists, stakeholders, and others interested in medical AI.

The NHS National AI Conference specifically was a combination of multiple lectures and a single workshop with shared access to exhibitions where attendees had hands-on experience with AI tools. It was led by three doctors based in the UK; two resident doctors and a former general practitioner, who is currently a digital health and innovation consultant. The conference aimed to educate health care professionals on defining AI, understanding its current uses and potential, the benefits and risks associated with integration in the NHS, and strategies to improve AI comprehension among the medical workforce. The conference was particularly targeted toward encouraging clinical professionals, such as doctors, surgeons, and nurses, to use AI.

### Learning objectives

Bloom’s taxonomy was used to create the following learning objectives aimed at enhancing participant understanding ^(15)^:

1. Define AI.

2. Establish an understanding of the benefits of AI in health care.

3. Critically analyze and evaluate risks associated with AI in health care.

4. Apply knowledge and understanding of medical AI to cultivate confidence and empowerment.

### Morning agenda

The morning agenda covered a variety of topics regarding AI in health care. This included multiple sessions showcasing current uses of AI in medicine and presentations such as “Understanding Healthcare Professionals’ Experience for Successful AI Adoption.” There were also two panel discussions. The first panel, “The Human Equations vs. the Algorithm: Shaping a Future of Care in the Smart Hospital,” explored how AI may improve clinical efficiency and patient experience in partnership with health care professionals. The second panel, “Healthcare Use Cases,” highlighted progression in the use of AI in the NHS and future trajectories.

### Afternoon agenda

The afternoon started with a workshop, “Empowering NHS Doctors: Harnessing AI and Digital Tools for Enhanced Healthcare.” This interactive workshop aimed to highlight ways doctors could use AI to improve patient care and apply AI in their regular clinical practice. This was followed by two further panel discussions. The “Trouble with AI in Healthcare” panel examined risks associated with AI, including bias, ethical concerns, liability, and how to limit these. The final panel, “Beyond Implementation: Building Healthcare’s AI-Fluent Workforce,” explored how to improve AI education among health care professionals to foster trust, enabling the operation of AI in clinical practice via a transparent curriculum.

### Ethics

Informed consent was obtained from all participants of this study through a presentation explaining the study, as well as a tick box within the survey confirming consent; our framework was based on Health Research Authority guidance ^(16)^. The UK Research and Innovation Medical Research Council and NHS Health Research Authority guidance cleared the study from formalized research ethics committee review ^(17)^.

### Data collection

An anonymized survey was distributed throughout the conference specifically to NHS professionals. Our survey was content-validated in accordance with the current literature on medical conferences as an educational intervention ^(18), (19)^, as well as the major pedagogical aims of medical AI education ^(10)^. We pre-tested our survey on a small cohort of NHS health care professionals. Divided into three sections, the first section collected data regarding attendee characteristics, such as their role and prior attendance to AI-related medical technology conferences. The second and third sections examined the impact of the conference on NHS professionals’ perceptions and understanding of AI through a before-and-after comparative study design. The survey was primarily composed of statements presented in a 5-point Likert scale, and a mixture of multiple-choice and short-answer open questions. Using paired questions, the survey aimed to assess how the conference influenced attendees’ understanding of current applications of AI in the NHS, associated benefits and risks, AI comprehension, and potential career ambitions.

### Data analysis

Statistical analysis was performed using R version 4.3.3. Frequencies were used to summarize questions on participant background as well as unpaired questions. For paired questions intended to ascertain changes pre-conference and post-conference, we trialed dichotomizing the data and performing McNemar’s test; however, we elected to use the Wilcoxon signed-rank test to better reflect the ordinal nature of the Likert-scale responses. Statistical significance was set at p < 0.05. Responses were then dichotomized with “Strongly agree” and “Agree” grouped to “Agree,” and “Strongly disagree,” “Disagree,” and “Neutral” grouped to “Neutral/Disagree.” A subgroup analysis was then performed with Firth’s logistic regression to calculate the odds of agreeing to each of the paired questions in participants who previously attended a MedTech AI conference or were clinicians, while controlling for participants who had previously agreed to the question.

## Results

There were approximately 3,000 attendees in 2024 cumulatively, and 54% of delegates were doctors. It is estimated that 100 attendees observed the NHS National AI Conference at any given time. The survey was completed by 43 attendees. As seen in Table 1, the majority (41.86%) of participants worked in the secondary health sector. Additionally, 53.49% were clinical professionals (37.2% were medical doctors, 4.65% were surgeons, 6.98% were nurses, and 4.65% were medical students). The majority of participants’ roles were encompassed under “Other,” which included data analysts, project managers, and medical device management leads. Most participants (65.12%) had not attended a medical technology conference before. Furthermore, the majority (62.79%) were unaware of how AI is used in the NHS at present.

**Table 1.** Participant parameters.

Parameter	Count (n = 43)	%
Healthcare provision region		
Primary	11	25.58
Secondary	18	41.86
Other	14	32.56
Healthcare role		
Medical doctor	16	37.21
Surgeon	2	4.65
Nurse	3	6.98
Medical student	2	4.65
Admin	1	2.33
Other	19	44.19

**Figure 3.**
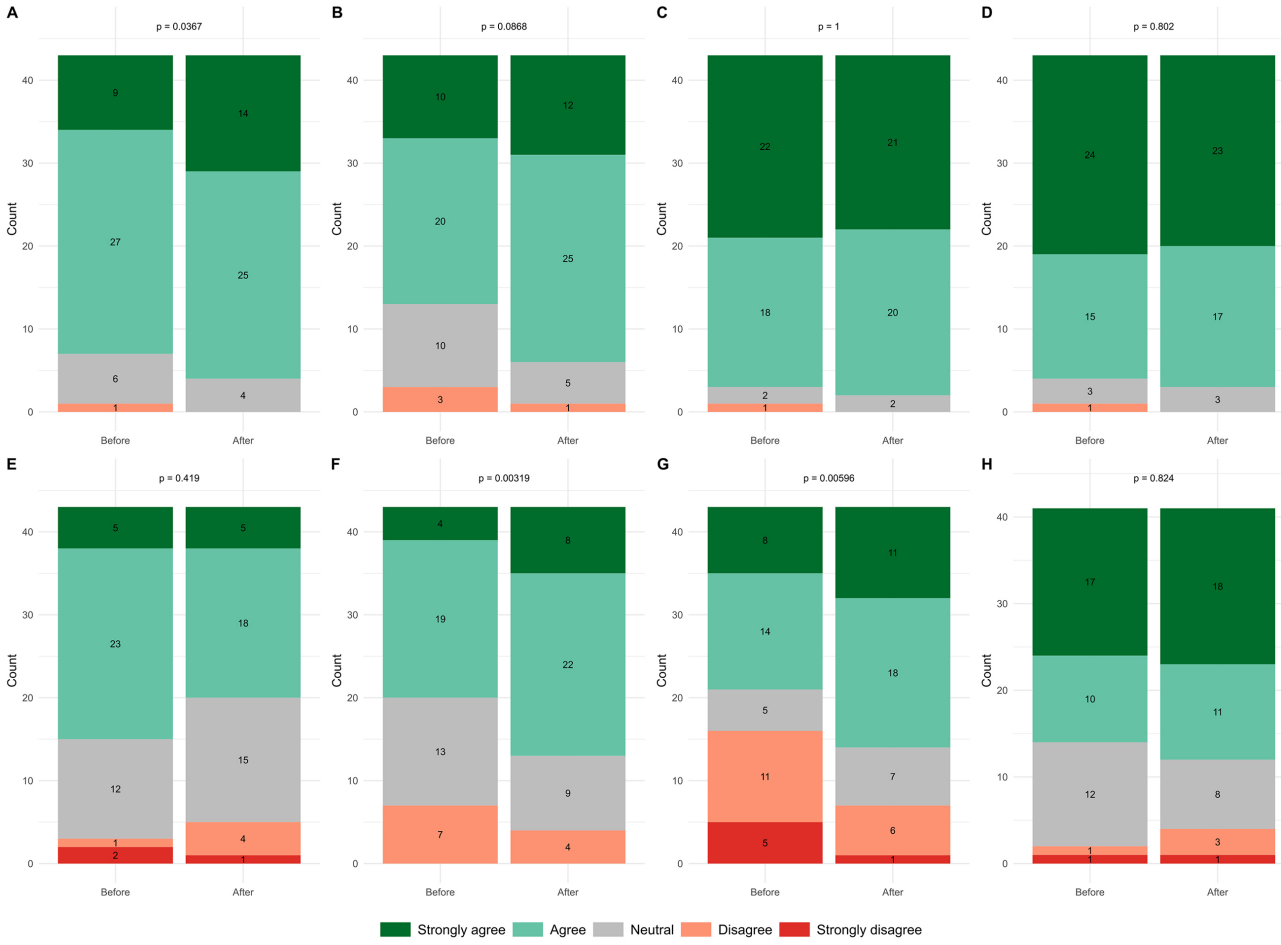
Stacked bar chart of responses to paired questions before and after the conference. p values are displayed for the Wilcoxon signed-rank test for each of the paired questions above the bar charts. From top down, each bar is divided into sub-bars for “Strongly Agree,” “Agree,” “Neutral,” “Disagree,” and “Strongly Disagree.” These are color-coded as described in the fill legend. A. I understand what artificial intelligence is. Before the conference, nine participants strongly agreed, 27 agreed, six were neutral, and one disagreed. After the conference, 14 participants strongly agreed, 25 participants agreed, and four were neutral (p = 0.0367). B. I understand the benefits/uses of artificial intelligence in health care. Before the conference, 10 participants strongly agreed, 20 agreed, 10 were neutral, and three disagreed. After the conference, 12 participants strongly agreed, 25 agreed, five were neutral, and one disagreed (p = 0.0868). C. AI and advanced technologies have the potential to improve patient care. Before the conference, 22 strongly agreed, 18 agreed, two were neutral, and one disagreed. After the conference, 21 strongly agreed, 20 agreed, and two were neutral (p = 1). D. AI and advanced technologies have the potential to improve hospital efficiency. Before the conference, 24 strongly agreed, 15 agreed, three were neutral, and one disagreed. After the conference, 23 strongly agreed, 17 agreed, and three were neutral (p = 0.802). E. I am concerned about the use of artificial intelligence in health care. Before the conference, five participants strongly agreed, 23 agreed, 12 were neutral, one disagreed, and two strongly disagreed. After the conference, five participants strongly agreed, 18 agreed, 15 were neutral, four disagreed, and one strongly disagreed (p = 0.419). F. I am confident using artificial intelligence. Before the conference, four participants strongly agreed, 19 agreed, 13 were neutral, and seven disagreed. After the conference, eight participants strongly agreed, 22 agreed, nine were neutral, and four disagreed (p = 0.00319). G. I feel empowered to use AI in my daily work. Before the conference, eight participants strongly agreed, 14 agreed, five were neutral, 11 disagreed, and five strongly disagreed. After the conference, 11 strongly agreed, 18 agreed, seven were neutral, six disagreed, and one strongly disagreed (p = 0.00596). H. I am interested in pursuing a career in medical AI. Before the conference, 17 participants strongly agreed, 10 agreed, 12 were neutral, one disagreed, and one strongly disagreed. After the conference, 16 participants strongly agreed, 11 agreed, eight were neutral, three disagreed, and one strongly disagreed (p = 0.824). AI: artificial intelligence.

Figure 3 summarizes the analysis of the paired questions. There was an increased understanding of AI after the conference among participants (p = 0.0367); this was statistically significant when analyzed with the Wilcoxon test, but McNemar’s test on dichotomized data did not reach statistical significance. Most participants (strongly agreed/agreed = 69.77%) understood the benefits/uses of AI before the conference. There was an increase in their understanding of the benefits after the conference (strongly agreed/agreed = 85.05%), although the Wilcoxon signed-rank test was not significant (p = 0.0868). Likewise, at baseline, most participants agreed that AI and advanced technologies have the potential to improve patient care (strongly agreed/agreed = 93.02% pre-conference vs. 95.35% post-conference, p = 1.00).

Most participants were concerned about the use of AI in health care before the conference (strongly agreed/agreed = 65.12%); this decreased to 53.49% of participants after the conference (p = 0.419). Concerns regarding medical AI were largely ethical, with 65.12% of participants highlighting this as their primary concern. Furthermore, 55.81% of participants were notably concerned about medical AI transparency, and 53.49% questioned its implementation.

Participant confidence in using AI was observed to increase from 53.49% to 69.77% (p = 0.00319). Furthermore, participants were observed to feel more empowered to use AI in their daily work (pre-conference 51.16% vs. post-conference 67.44%, p = 0.00596). For both confidence and empowerment, the Wilcoxon test―but not McNemar’s test on dichotomized data―reached statistical significance. Finally, interest in pursuing a career in medical AI increased from 62.79% to 67.44% but did not reach statistical significance (p = 0.824).

**Figure 4.**
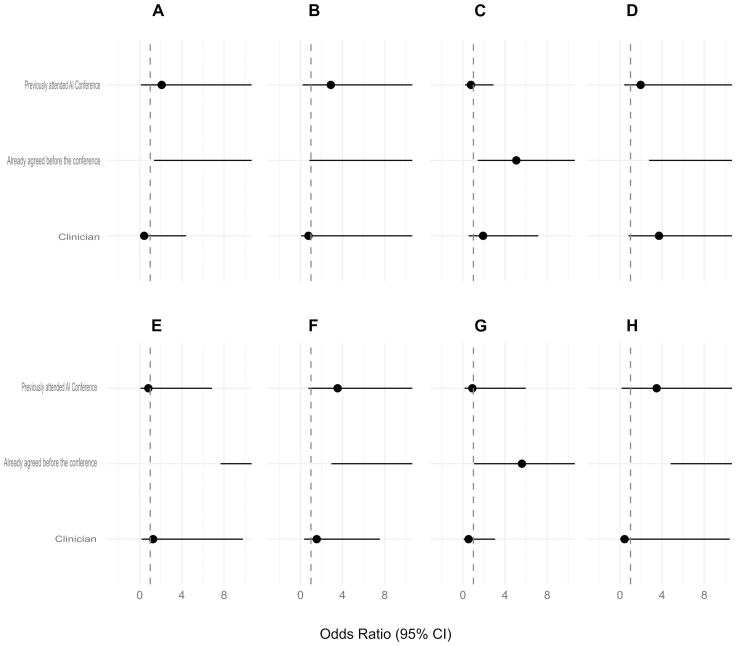
Forrest plots displaying the adjusted odds ratios of agreeing to each of the questions after the conference for participants who previously attended a MedTech AI conference or were clinicians, while controlling for previous agreement to each respective question. This was generated through separate logistic regression models for each question. The x-axis represents the odds ratio. On each graph, the solid dot represents the odds ratio, with a horizontal line representing the 95% confidence interval. Due to scale, the confidence intervals are not fully demonstrated. The y-axis found on plots A and E denote the labels for each predictor for plots A, B, C, D and plots E, F, G and H respectively. A. I understand what artificial intelligence is. B. I understand the benefits/uses of artificial intelligence in health care. C. AI and advanced technologies have the potential to improve patient care. D. AI and advanced technologies have the potential to improve hospital efficiency. E. I am concerned about the use of artificial intelligence in health care. F. I am confident using artificial intelligence. G. I feel empowered to use AI in my daily work. H. I am interested in pursuing a career in medical AI. For all questions, when controlling for agreement in the pre-conference questionnaire, previously attending an AI conference and being a clinician did not have a statistically significant impact on any of the paired questions. AI: artificial intelligence.

Our subgroup analysis, using Firth’s logistic regression, showed no significant association between being a clinician or having previously attended a MedTech AI conference on improvements in any of our paired questions (Figure 4).

## Discussion

This study evaluated the effectiveness of a dedicated medical AI conference, the NHS National AI Conference held within the larger GIANT Health event, as a tool to improve medical AI comprehension among health care professionals. Employing a before-and-after survey design, we assessed changes in attendees’ perceptions and understanding of AI in health care.

Our findings, based on a survey completed by 43 attendees, suggest a positive trend toward improved medical AI comprehension among health care professionals. Participants were largely optimistic about its ability to improve efficiency (strongly agreed/agreed = 90.70% pre-conference) and patient outcomes (strongly agreed/agreed = 93.02% pre-conference). This indicates a positive perception of clinical AI at baseline, which is reflected in an international two-stage cross-sectional survey on AI attitudes among the neurosurgical team, which found that 85% of 100 health care professionals, including surgeons, anesthetists, and nurses, considered alerts to be valuable for the early detection of complications ^(20)^. Our study observed a 15.28% increase in participants’ understanding of the benefits and applications of AI in health care after the conference that did not reach statistical significance. Furthermore, there was an increase in empowerment among participants in using AI in their daily work (p = 0.00596) that reached statistical significance using the Wilcoxon, but not McNemar, test. This suggests a potential readiness among health care professionals to implement AI in their practice following an understanding of its benefits. This is supported by a systematic review by Gazquez-Garcia et al. ^(10)^ in Spain, which concluded that understanding propagates the efficient implementation of medical AI. Ultimately, enhancing health care professionals’ comprehension of AI’s advantages in their respective fields may increase engagement with AI and maximize its potential, foster innovation, and encourage sustainable improvements within the health care system.

The proportion of participants concerned about the use of AI in health care decreased following the conference (strongly agreed/agreed = 65.12% pre-conference vs. 53.49% post-conference), although this was not statistically significant. This may be due to an increased understanding of AI’s benefits, as suggested by our findings. Indeed, a systematic review of 43 studies by Ahmed et al. ^(9)^ highlighted trust as a barrier for AI implementation. Concerns about trust among health care workers revolved around transparency, ethical considerations, and implementation challenges. Transparency, in particular, is key in areas such as financial concerns, ethical dilemmas, data security, and ambiguity around liability ^(9)^. We postulate that transparent communication about the risks and benefits of AI may empower health care professionals to self-evaluate new software and subsequently build trust by decreasing their fear of the unknown.

Furthermore, participant confidence in using AI (p = 0.00319) and empowerment in using AI in their daily work (p = 0.00596) were observed to have increased, reaching statistical significance when not dichotomized and analyzed using the Wilcoxon test. This suggests that the conference may have started to address skill gaps presently limiting AI adoption ^(10)^. These improvements are encouraging for the potential integration of AI tools into clinical workflows. However, considering that attendees at conferences typically have a fundamental understanding of AI (83.72% pre-conference), future conferences must extend beyond introductory concepts, exploring more advanced topics and creating in-depth discussions. As recommended by Gazquez-Garcia et al. ^(10)^, AI education should encompass five vital competencies, including fundamentals, ethics, data analysis, communication, and evaluation ^(10)^. This could be expanded by covering the practical uses of AI in health care, data and business management, regulatory challenges, security, and future applications.

There was no significant increase in the understanding of benefits/uses of AI in health care (p = 0.0868), nor was there a significant change in concern (p = 0.419). This highlights the need for new educational interventions. Specifically, workshops may be designed to establish a deeper understanding of AI by exploring its use and evaluating existing technologies. These workshops should further illustrate the benefits and limitations of using AI in clinical settings, providing participants with opportunities to use and refine their skills, troubleshoot in simulated scenarios, and further drive the adoption of AI in medical practice. In a study by Blake and Das ^(6)^, clinicians working in East Kent University Hospitals received a workshop series to train them on the use of a deep learning algorithm implemented in the department to report chest X-rays. Seventy-nine percent of workshop participants felt the algorithm would decrease missed findings. After implementation, clinicians reported mistakes, and improvements were made to the algorithm; consequently, sensitivity was 99% ^(6)^. Ultimately, this highlights that workshops empower clinicians with the skills and knowledge required to actively question AI and troubleshoot, which creates increasingly accurate algorithms and maximizes AI’s potential in health care.

There was an observed increase in participants who felt empowered (p = 0.00596) to use AI in their daily work after the conference; this reached significance when data were analyzed without dichotomization. Increased empowerment may drive health care workers to integrate AI into their clinical practice and engage with further training. However, how AI education is integrated is paramount in maintaining empowerment. In a qualitative study by Ganapathi and Duggal, doctors expressed concern regarding the lack of time available outside their official duty hours for additional courses on AI, which would add to the already overwhelming workload ^(21)^. In fact, 89.4% of participants believed that they would have an increased workload when using AI ^(22)^. This is supported by an observational study surveying radiographers, which demonstrated that 43.4% had received AI training via a wide range of agencies outside of their formal education ^(23)^. Consequently, not only will this produce discrepancies between professionals and their understanding, but it also highlights the current issue of seeking training outside of contracted hours, thus increasing workload and creating costs to facilitate AI integration. Therefore, incorporating courses on AI into the ongoing mandatory training expected of health care workers would be a natural extension of the current educational model. It may alleviate concerns regarding an increased workload and standardize AI education. This fosters a culture of continuous learning and improvement, which may further drive innovation by encouraging collaboration.

While our study suggests a positive trend toward improved medical AI comprehension among health care professionals, a larger sample size would enhance the statistical power of our results. The sampling fraction could be increased in future studies by including survey completion as part of participant certificate provision. More in-depth workshops instead of multiple panel discussions may improve understanding of the benefits and uses of AI. Additionally, the inclusion of a diverse cohort of health care professionals may have limited our conference’s ability to provide AI education specific to their role. For example, it is likely that our participants will interact with AI in different ways; administrative staff may benefit from automated bureaucratic tasks, in contrast to doctors, for whom clinical alerts or AI radiology reporting may be more valuable ^(6), (7), (23)^. Indeed, the inconclusive nature of our subgroup analysis limits our ability to recommend a particular professional subgroup to be targeted by future conferences. Future studies should consider further stratifying baseline knowledge levels or target groups to better identify responsive subgroups. Furthermore, our study focused on attendees of a single conference in the UK; a future multinational study design would confirm international generalizability. Finally, our study focused on quantitative analysis and collected limited qualitative data; future studies could look to include qualitative evaluation to enhance understanding of participants’ learning experiences.

To facilitate the successful integration of AI into clinical practice via improved AI literacy, we propose several key recommendations. First, the implementation of a national, standardized AI curriculum is imperative to equip health care professionals with the skills needed for AI integration. This curriculum should be incorporated into both undergraduate and postgraduate medical education, as well as continuous professional development programs to ensure a consistent baseline of AI literacy regardless of training pathway or employing organization ^(24)^. Moreover, curriculum standardization mitigates the need for repeated retraining when professionals switch between employers.

Second, robust data security frameworks and transparent governance policies must be established in order for health care professionals to feel confident that AI using confidential patient data is secure. National guidelines and protocols regarding data security, aligned with regulations such as the UK General Data Protection Regulation, are essential to ensure a trustworthy environment for AI utilization ^(23)^. A national standardization of data governance will improve educational efforts by increasing trust and therefore willingness to engage in learning.

Finally, to complement a national AI curriculum and standardized data governance, strategic investment in a modern and interoperable information technology (IT) infrastructure is essential to fully realize the benefits of an AI-literate workforce. While education equips health care professionals with the knowledge to utilize AI effectively, the current inadequacy in IT infrastructure across NHS trusts presents a significant barrier to the practical application of this knowledge ^(7), (24)^. A national strategy for IT procurement could improve interoperability between and within trusts. This will ensure effective implementation of AI-driven solutions and will maximize the return on investment in AI education by providing a consistent and functional technological landscape for its application in improving patient care and health care efficiency.

In conclusion, our study suggests medical AI conferences may enhance AI comprehension among health care professionals. We observed potential improvements in AI understanding, participant confidence, and participant empowerment to use AI in daily work, although further robust studies are required to establish the generalizability of our findings. We observed a baseline optimism regarding medical AI; this presents a potentially receptive environment for further education and integration efforts. With health care services worldwide integrating medical AI tools, accessible and standardized national AI curricula and a supportive technological infrastructure will be crucial to realizing the potential of a motivated workforce to drive innovation and improve patient care. Future conferences could form a part of these national curricula that standardize skills and knowledge across the workforce. These may be run locally and integrated into broader national strategies. This should be complemented by practical, skill-based learning opportunities, such as interactive workshops that enable hands-on experience and critical evaluation of AI tools. Addressing concerns regarding ethics, transparency, and workload is a necessity to foster trust and improve AI comprehension and consequently, integrate AI into health care systems. Future research should adopt both quantitative and qualitative approaches, utilize larger and more stratified samples, and include performance-based measures to evaluate not only self-reported understanding but also practical competence in medical AI. Establishing interventions within clearly defined evaluation frameworks will further improve the rigor and interpretability of research in this field. By investing in structured educational initiatives and cohesive policy frameworks, health care systems may accelerate the responsible and effective adoption of AI―ultimately enhancing patient care and operational efficiency on an international scale.

## Article Information

### Author Contributions

We confirm that authors Nafisa Islam, Abdel Rahman Osman, and Laveinia Godfrey all contributed equally to this work.

### ORCiD iD

Nafisa Islam: https://orcid.org/0000-0002-7459-0467

Abdel Rahman Osman: https://orcid.org/0000-0001-8244-9474

### Conflicts of Interest

None

### IRB Approval Code and Name of the Institution

None.
